# Distal Humeral Fractures-Current Concepts

**DOI:** 10.2174/1874325001711011353

**Published:** 2017-11-30

**Authors:** James C. Beazley, Njalalle Baraza, Robert Jordan, Chetan S. Modi

**Affiliations:** University Hospitals Coventry and Warwickshire NHS Trust, Clifford Bridge Road, CV2 2DX, Coventry, UK

**Keywords:** Distal humerus fracture, Fracture fixation, Open reduction internal fixation, Total elbow arthroplasty, Anatomy, Elbow

## Abstract

**Background:**

Distal humerus fractures constitute 2% of all fractures in the adult population. Although historically, these injuries have been treated non-operatively, advances in implant design and surgical technique have led to improved outcomes following operative fixation.

**Methods:**

A literature search was performed and the authors’ personal experiences are reported.

**Results:**

This review has discussed the anatomy, classifications, treatment options and surgical techniques in relation to the management of distal humeral fractures. In addition, we have discussed controversial areas including the choice of surgical approach, plate orientation, transposition of the ulnar nerve and the role of elbow arthroplasty.

**Conclusion:**

Distal humeral fractures are complex injuries that require a careful planned approach, when considering surgical fixation, to restore anatomy and achieve good functional outcomes.

## 1 INTRODUCTION

Distal humerus fractures constitute 2% of all fractures in the adult population [1]. The injuries are distributed in a bi-modal fashion with the first peak being seen in the young resulting from high-energy trauma and the second peak being seen in the elderly osteoporotic population [2]. Although relatively rare, the incidence of these fractures is rising as Pavlanen *et al.* reported a 5 fold increase in distal humerus fractures between 1970 and 1998 [3]. Treatment is aimed at restoring a functional elbow, which Morrey described as requiring 30 to 130 degree range of motion [4]. Loss of this movement can severely affect activities of daily living and lead to a loss of independence in the elderly population [5]. Treatment of these injuries is challenging due to fracture comminution, poor bone quality and difficulty in restoring the complex anatomy of the distal humerus.

Historically, these injuries have been treated non-operatively although most studies report this management to be associated with significant functional impairment [2]. Evolution in implant design and surgical technique has led to improved outcomes in operatively treated patients and has resulted in fixation being the current standard of care. Operative fixation has been shown to give satisfactory results with long term follow up demonstrating good or excellent outcome in 86% [6, 7]. In an elderly population, internal fixation has been reported to result in better function than those managed non-operatively [8]. The goals of surgical treatment are to restore articular congruity and bone alignment whilst providing rigid, stable fixation that enables early active motion [9, 10]. Currently controversy exists over several issues of operative management including the optimal surgical approach, plate orientation, management of the ulnar nerve and the role of elbow arthroplasty. This review will cover the anatomy and classification of distal humerus fractures, investigations and the treatment options for distal humerus fractures including discussion surrounding these controversial areas.

## 2 ANATOMY AND CLASSIFICATION OF DISTAL HUMERAL FRACTURES

In the coronal plane, the distal humerus is of triangular shape, formed by the medial and lateral columns linked by the articular segment as illustrated in Fig. (**1**). This simple concept is useful when trying to reconstruct complex fractures as described by O’Driscoll [11]. The central area is thinner and compromises the coronoid fossa and olecranon fossa. The articular surface is in 4-8 degrees of valgus relative to the shaft and flexed 40 degrees relative to the shaft. The AO classification is the most commonly used classification system. Fractures are divided into A-extra-articular, B-partial articular and C-complete articular, with further subdivision of these groups into 1,2, and 3 depending on the location of the fracture and the degree of comminution.

## 3 IMAGING DISTAL HUMERAL FRACTURES

Good quality radiographs should be obtained although this may be difficult due to patient discomfort. For the distal humerus, the AP is taken with the elbow flexed to 40 degrees to facilitate olecranon disengagement from the fossa allowing for better visualisation of the distal humerus. Standard lateral images, with the shoulder abducted to 90 degrees and the elbow flexed to 90 degrees with the plate underneath the medial aspect give good views of the humeral condyle and olecranon. A CT scan can give further clarity regarding comminution, articular involvement and aid surgical planning. At our institution, we obtained 3D reconstructions of all intra-articular distal humerus fractures to aid our pre-operative planning. This practice is supported by evidence to suggest that the use of 3D reconstructions over 2D images improves the accuracy of classification [12].

## 4 NON-OPERATIVE TREATMENT

Non-operative treatment involves temporary splintage for pain relief followed by gentle mobilisation. This approach has been associated with poor outcomes. Nauth *et al.* demonstrated that in elderly patients, those treated non-operatively were almost three times more likely to have an unacceptable result than those treated operatively (RR=2.8 95% CI 1.78-4.4) [13]. Non-operative management is consequently reserved for undisplaced fractures and for patients with dementia or those unable to tolerate anaesthesia. Recently Aitken *et al.* reported that non-operative treatment could give a modest functional result in low demand patients whilst avoiding the substantial surgical risks [14].

## 5 OPERATIVE INTERVENTION FOR DISTAL HUMERUS FRACTURES

### 5.1 Surgical Approach

Numerous surgical approaches to the distal humerus have been described, each conferring differing advantages in terms of exposure and soft tissue disruption. Stanley demonstrated that the trans-olecranon approach gave the best articular exposure; the median percentages of visible distal humeral articular surface for the triceps splitting, triceps reflection, and olecranon osteotomy approaches were 35%, 46% and 57%, respectively [15]. Despite the olecranon osteotomy providing the greatest exposure of the distal articular surface, 40% of the anterior surface remained unvisualised [15]. In addition, an olecranon osteotomy carries the risk of non-union, future need for the removal of metalwork and potentially limiting any future arthroplasty.

The triceps splitting approach was first described by Campbell [16] but has the potential to result in triceps weakness. Bryan and Morrey described a triceps reflecting technique [17] that spares the triceps mechanism by reflecting from medial to the lateral direction and has the advantage of avoiding damage to the extensor mechanism. At our institution, for management of an extra-articular fracture (AO type A) or limited articular involvement (type B1/B2), we utilise the triceps sparing approach. This approach is chosen as it gives adequate exposure for these fractures, avoids disruption to the extensor mechanism and can still be converted to an olecranon osteotomy if greater exposure of the articular surface becomes required. For complex intra-articular fractures (type C), an olecranon osteotomy is preferred.

### 5.2 Olecranon Osteotomy Technique

The patient is positioned in the lateral position with the arm hanging over an “L” holder. The image intensifier is positioned such that it can be easily moved in and out of the operative field with adequacy of imaging being checked prior to prepping and draping of the patient. The arm is exsanguinated and a sterile tourniquet inflated. A midline posterior incision is made being proximal to the level of the fracture and curving down lateral to the olecranon avoiding the weight-bearing zone. Full-thickness medial and lateral flaps are created. The ulnar nerve is identified proximally and released from the cubital tunnel and through the flexor pronator aponeurosis to the level of its first anterior motor branch.

The interval between the medial intermuscular septum and triceps is developed. The triceps is elevated off the posterior aspect of the humerus following which, the lateral window is created. If the dissection is extended proximally, the radial nerve is encountered approximately 10cm proximal to the lateral epicondyle and should be sought and protected. A capsulotomy is performed on either side of the olecranon and the “bare area,” of the ulna is identified. The apex distal chevron osteotomy is performed with an oscillating saw and completed with an osteotome to facilitate interdigitation. A swab can be used to protect the articular surface when completing the osteotomy. The olecranon fragment is then wrapped in a saline soaked swap and sutured proximally thus completing the approach.

### 5.3 Fracture Fixation

The goal of fixation is to provide anatomic reduction and rigid internal fixation of the articular surface to the shaft to allow early mobilisation. The use of fixation solely with Kirschner wires or screws has been shown to be inferior to rigid plate fixation in terms of functional outcome [18]. Although plate fixation in now the standard treatment, there remains a debate on the optimum configuration of plates. Perpendicular plating was originally advocated by the AO group, but parallel plating has grown in popularity. Biomechanical studies have demonstrated that parallel plating provides improved stability compared with the perpendicular locking system [3, 19-21]. However, clinical studies that have compared perpendicular to parallel plating reported no difference between the groups in terms of functional outcome or complication rate [22, 23]. The use of locking compression plates has been advocated for osteoporotic fractures as they provide angular stability and the head-locking mechanism potentially results in a stiffer construct [24]. However, biomechanical studies have shown no significant difference in stiffness between locking and traditional compression plates [25]. Therefore although fixed angle plates have gone some way to improving fixation in the presence of poor bone stock, loss of fixation in the osteoporotic patient remains a concern [26].

Surgical technique is dependent on the fracture pattern but O’Driscoll described eight key surgical principles to base operative fixation upon (Table **1**). Following the approach the fracture surfaces are gently debrided. The least comminuted column is reduced and held with bone clamps and temporary Kirschner wires. This allows accurate reconstruction of the more comminuted parts including the articular surface. Subchondral screws may sometimes be required to reconstruct articular fragments. Two plates are utilised with the less comminuted column selected first and secured with a pre-contoured locking plate before the second column is then fixed with a further pre-contoured plate. Different length plates are chosen to avoid a stress riser at the end of the plates and risk of a peri-prosthetic fracture. An example case of operative fixation is demonstrated in Figs. (**2** and **3**). At our centre typical post-operative management protocol includes; a back slab for one to two weeks to protect the wound, passive mobilisation from two weeks then active after six weeks.

Despite the evolution of surgical techniques, operative fixation has been associated with dissatisfaction in 15% of patients and complication rates of up to 35% [27, 28]. Known factors that affect outcome include fracture comminution, reduction accuracy, fixation stability and quality of postoperative rehabilitation [29]. Complications can include hardware failure, fracture, non-union, malunion, heterotopic ossification, elbow stiffness and ulnar neuropathy [30-33]. An example of a peri-prosthetic fracture is illustrated in Fig. (**4**).

The incidence of heterotopic ossification (HO) following fixation of distal humerus fractures varies widely and is reported to be between 0 and 21% [34]. Risk factors for HO include head injury, delay in operative intervention, and surgery prior to definitive fixation. To date there is no level 1 evidence examining the role of HO prophylaxis in the management of distal humerus fractures treated with ORIF. Nauth *et al*. pooled the results of 6 studies examining the incidence of HO in 259 patients undergoing fixation of distal humeral fractures utilising modern fixation techniques and not treated with HO prophylaxis [13]. They found an 8.6% rate of symptomatic HO. Two recent case series have both reported HO rates of 3% in 67 patients with distal humerus fractures treated with HO prophylaxis, but did report a non-union rate of 6% [22, 35]. At our institution we do not routinely give HO prophylaxis unless risk factors are present.

Three-quarters of malunion or non-union cases are caused by inadequate initial fracture fixation suggesting that optimal stable fixation is difficult to achieve [36]. Elderly patients are particular at risk of fixation failure [37, 38] and John *et al.* reported 26% of over 80 year old’s were dissatisfied with the outcome following surgery [39]. This has led to the introduction of total elbow arthroplasty in certain patients but this remains a controversial area.

## 6 ELBOW ARTHROPLASTY

Total elbow arthroplasty (TEA) is becoming recognised as a safe and effective alternative to operative fixation in the treatment of comminuted intra-articular distal humerus fractures in the elderly patient [22]. This is reflected in the number of TEAs performed annually for distal humeral fractures, which has increased 2.6 fold between 2002 to 2012 [34]. An example of a case managed with TEA is illustrated in Figs. (**5** and **6**). TEA has limited longevity due to aseptic loosening, an example of which is illustrated in Fig. (**7**), therefore TEA is only recommended in patients with sedentary lifestyles [40] who can comply with the post-operative rehabilitation regime [22]. TEA patients will have lifelong restrictions placed upon them with limited weight bearing to decrease risk of premature wear or subsequent revision [41]. Prasad *et al*. [42] analysed the survival of TEA in fracture patients and showed that only 53% had implant survival of more than 10 years and 89.5% of patients demonstrated loosening of their prostheses at this stage.

McKee *et al.* compared operative fixation with TEA in a prospective, randomized, multicenter study for 40 patients presenting with comminuted, displaced, intra-articular fractures of the distal humerus (OTA/AO type C) in patients over the age of 65 years [33]. The study reported better Mayo Elbow Performance Score in the total elbow arthroplasty group at 6 months (86 vs 68, P = .003), 12 months (88 vs 72, P = .007), and 2 years (86 vs 73, P = .015). A significant difference favoring DASH score was reported at 6 months but at no further time point. Additionally there was a 25% rate of intra-operative conversion to TEA in the ORIF group because of extensive comminution and an inability to achieve stable fixation. Reoperation rates for TEA (3/25 [12%]) and ORIF (4/15 [27%]) were not statistically different (P = 0.2). The authors concluded that TEA is a preferred alternative to fixation in elderly patients with complex distal humeral fractures that are not amenable to stable fixation [33]. In a recent systematic review comparing TEA to ORIF for the treatment of distal humeral fractures in the elderly, Githens et. al. pooled the results of 27 papers including 563 patients and found no significant difference between groups with respect to functional outcome or complications rates [41]. Currently this area and the treatment of choice for these patients remain unclear.

Hemiarthroplasty is an alternative option and has the theoretical advantage of reducing the polyethylene wear and being a more durable implant [43, 44]. However for implantation the patient must have an intact or reconstructable radial head, coronoid, medial and lateral columns and functional collateral ligaments [45]. The implant requires fewer restrictions on the patients’ activities and so may be a feasible option for slightly younger patients. Good outcomes have been demonstrated in small case series with medium term follow up [45, 46], although reported complication rates are comparable to that of TEA [42].

## 7 ULNAR NERVE

The ulnar nerve should be identified and protected throughout the surgical procedure. Controversy surrounds the benefit of routine ulna nerve transposition following fixation; transposition theoretically lets the ulnar nerve lie in a fresh bed but given the need to dissect further the nerve may become devascularised [47]. Chen *et al.* performed a retrospective review analysing the results of 89 patients undergoing ORIF for distal humerus fractures who underwent either a decompression alone or combined with transposition. The authors reported a significantly increased rate of ulna neuritis post-operatively in the transposition group vs. the non-transposition group (33% vs 9% p = 0.0003) and concluded there was no clear benefit to ulna nerve transposition [48]. For patient with pre-operative ulnar nerve symptoms then transposition has been reported to improve recovery of symptoms (p <0.05) [49]. At our institution we do not routinely transpose the nerve unless significant subluxation of the nerve exists on flexion following reconstruction.

## 8 CORONAL SHEAR FRACTURES

Capitellar fractures are rare accounting for only 1% of total elbow injuries and 6% of distal humeral fractures [50]. The mechanism of injury is usually resulting from a fall on an outstretched hand or flexed elbow where radial head imparts a shear force on the capitellum, lateral trochlear ridge and trochlea [51]. Isolated coronal shear fractures of the capitellum or trochlea (AO type B3) are relatively rare injuries and account for a small percentage of distal humerus fractures [52]. Coexisting soft tissue or bony injuries are common with 60% of patients with coronal shear fractures also having lateral collateral ligament (LCL) injuries or radial head fractures [53]. The most commonly used classification for these fractures is the Bryan and Morrey classification system [54]. It is based on 3 types of capitellum fractures. Type 1 (Hahn-Steinthal) lesions involve a fracture isolated to the capitellum with attached subchondral bone. Type 2 fractures (Kocher-Lorenz) predominantly involve the articular cartilage overlying the capitellum. Type 3 (Broberg-Morrey) lesions are comminuted capitellum fractures. A fourth type was later added by McKee that is defined as a capitellum fracture that extends medially into the trochlea [51].

Inadequately treated capitellum fractures may lead to poor function and reduced range of motion [55, 56]. Poor results have been reported with conservative management of these fractures but are also associated with posterior comminution of the lateral condyle or multiple articular fragments [57]. Excision of the capitellar fragment has been suggested [58, 59]; however, in the long run, this will lead to radiohumeral osteoarthritis and instability of the elbow [60, 61].

Typically the lateral approach is used to gain access to the fracture. In the presence of an incompetent LCL, the elbow can be hinged open, gaining good exposure to the joint surface. If the LCL is intact, either the plane between the extensor digitorum communis and extensor carpi radialis brevis (Kaplan interval – the author’s preference) or the more posterior plane between the anconeus and extensor carpi ulnaris (Kocher interval) can be used to access the fracture [51, 54]. The fracture is fixed with anterior to posterior headless variable pitch screws; these implants have been shown to be superior to either Kirschner wires or cancellous screws [62]. In the presence of metaphyseal comminution, a lateral plate may be applied. The LCL is then repaired if incompetent. Patients are placed in a back slab for two weeks to protect the wound and are then fully mobilised.

## CONCLUSION

Distal humerus fractures are a challenging but rewarding group of injuries to manage. Advances in plate design and surgical technique have improved the outcome for patients undergoing internal fixation, which is the current standard treatment. However the procedure can be challenging and has a high rate of complications. Total elbow arthroplasty has been advocated as a treatment option especially for elderly patients or those with unreconstructable distal humerus fractures.

## Figures and Tables

**Fig. (1):**
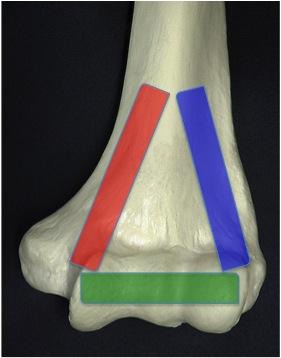
Distal humerus triangle. Triangle formed by medial column (red), lateral column (blue) and articular surface (green).

**Fig. (2):**
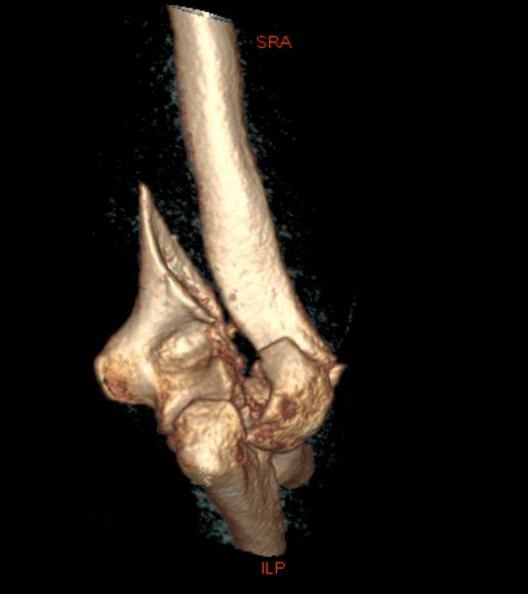
Pre-operative CT and parallel plating of AO type C1 fracture.

**Fig. (3):**
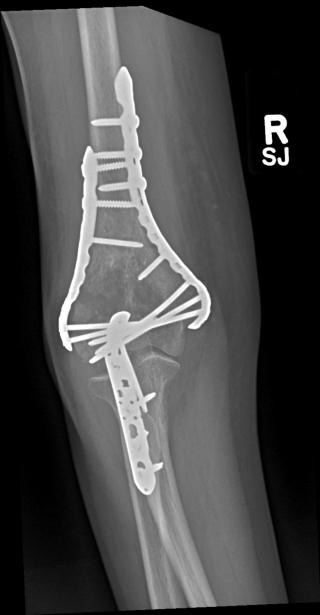
Pre-operative CT and parallel plating of AO type C1 fracture.

**Fig. (4):**
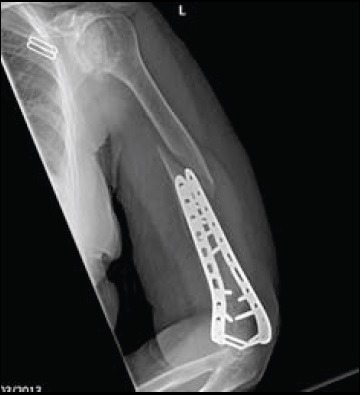
Example of peri-prosthetic fracture following a mechanical fall one year after fixation of distal humerus.

**Fig. (5):**
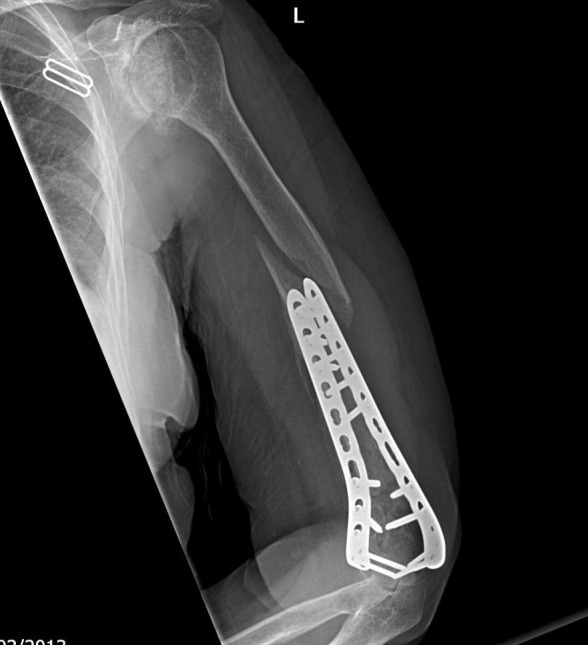
Comminuted distal humerus fracture (AO type C3) in an elderly patient undergoing TEA.

**Fig. (6):**
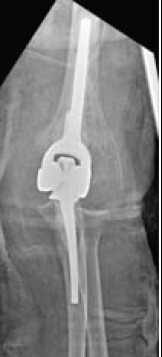
Comminuted distal humerus fracture (AO type C3) in an elderly patient undergoing TEA.

**Fig. (7):**
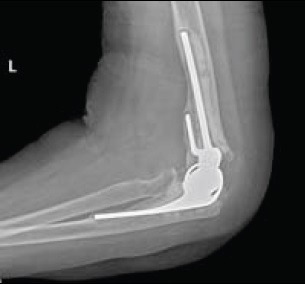
Example of loosening of elbow arthroplasty.

**Table 1:** Technical objectives described O’Driscoll [11].

Sr.No	**Objective**
1	Every screw should pass through a plate
2	Each screw should engage a fragment on the opposite side that is also fixed to a plate.
3	As many screws as possible should be placed in the distal fragments.
4	Each screw should be as long as possible.
5	Each screw should engage as many articular fragments as possible.
6	The screws should lock together by interdigitation within the distal fragment, thereby creating a fixed-angle architecture that provides stability to the entire distal humerus.
7	Plates should be applied such that compression is achieved at the supracondylar level for both columns.
8	Plates used must be strong enough and stiff enough to resist breaking or bending before union occurs at the supracondylar level.
